# Modification of episodic memories by novel learning: a failed replication study

**DOI:** 10.1080/20008198.2017.1315291

**Published:** 2017-05-16

**Authors:** Kevin van Schie, Suzanne C. van Veen, Marcel A. van den Hout, Iris M. Engelhard

**Affiliations:** ^a^ Department of Clinical Psychology, Utrecht University, Utrecht, The Netherlands

**Keywords:** Memory reconsolidation, episodic memory, replication, Bayesian statistics

## Abstract

**Background**: After reactivation, memories can become unstable and sensitive to modification before they are restored into long-term memory. Using behavioural manipulations, reactivated memories may be disrupted via the mechanism of interference (i.e. novel learning). In a laboratory study, Wichert et al. (2013a) showed that new learning after reactivation changed episodic memory, while new learning alone or reactivation alone did not.

**Objective:** Given the potential clinical application of such a procedure in trauma-focused psychological treatments, such as CBT or EMDR, the aim of this study was to replicate Wichert et al.

**Method**: On Day 1, participants (*N* = 96) viewed and recalled a series of emotional and non-emotional pictures. Then, participants were randomized to one of four groups. One week later, on Day 8, Group 1 reactivated the previously learned pictures and learned new pictures. To control for specific effects of reactivation or new learning, Group 2 only reactivated the previously learned pictures, and Group 3 only learned new pictures. Group 4 received no reactivation and no new learning. On Day 9, all groups indicated for each picture out of a series whether they had seen it on Day 1.

**Results**: The data were analysed using Bayesian hypothesis testing, which allows for quantifying the evidence in favour of the alternative and the null hypothesis. In general, results showed that Group 1 recognized fewer pictures from Day 1 compared to Groups 2 and 4 on Day 9. However, the expected difference between new learning following reactivation (i.e. Group 1) and new learning alone (i.e. Group 3) was not substantially supported by the data for any of our dependent measures.

**Conclusions**: We replicated some of the findings by Wichert et al., but did not find substantial support for the critical difference between new learning following reactivation and new learning alone.

For the past 20 years, psychological science has seen a fast-growing interest in memory reconsolidation (Nader, 2015). Memory reconsolidation is the process in which reactivated, consolidated memories require a stabilization phase during which they are temporarily sensitive to amnesic agents (for an overview see Ågren, 2014; Besnard, Caboche, & Laroche, 2012; Schwabe, Nader, & Pruessner, 2014). In cognitive psychology, it has long been known that episodic memories are malleable (e.g. Loftus & Palmer, 1974), but, prior to their groundbreaking work on reconsolidation (e.g. Nader, Schafe, & LeDoux, 2000), behavioural neuroscientists believed that emotional memories were indelible (LeDoux, Romanski, & Xagoraris, 1989). A seminal study by Nader et al. (2000) brought these two research disciplines closer by demonstrating that consolidated memories can indeed be changed. Nader et al. showed that memories are impaired when the reconsolidation process is disrupted, by injecting rodents with a pharmacological agent (i.e. anisomycin) shortly after reactivation of consolidated memories. Because this agent blocks the protein-synthesis that is necessary for long-term memory formation, there was amnesia for these memories.

Reconsolidation research was extended quickly from animals to humans and started focusing on two methods of reconsolidation manipulation to test the boundary conditions of reconsolidation (Ågren, 2014). One line of research remained close to the animal studies and demonstrated experimentally that human memories can be changed via pharmacological manipulations (e.g. Brunet et al., 2008; Kindt, Soeter, & Vervliet, 2009; Sevenster, Beckers, & Kindt, 2012, 2013; Soeter & Kindt, 2012), while the other line showed that memories can be altered with behavioural manipulations (e.g. Forcato et al., 2007; Hupbach, Gomez, Hardt, & Nadel, 2007; James et al., 2015; Schwabe & Wolf, 2009; Schiller et al., 2010; Wichert, Wolf, & Schwabe, 2011; 2013a, 2013b; see also van den Hout & Engelhard, 2012). Using behavioural manipulations, reactivated memories can be updated or disrupted during reconsolidation via novel learning (Ågren, 2014). An example of such a study is the one by Wichert et al. (experiment 1, 2013a), who used the canonical three-day design and normative emotional stimuli. On Day 1, all four groups viewed and recalled a series of pictures (i.e. Set 1). On Day 8, one week later, one of four groups reactivated the previously learned pictures and learned new pictures (i.e. Set 2). To control for specific effects of reactivation or new learning, a second group only learned new pictures without reactivation, and a third only reactivated the previously learned pictures without new learning. A fourth group received neither reactivation nor new learning. On Day 9, all groups performed a recognition test in which they classified whether pictures from Set 1 and Set 2, and a set of pictures that were never seen (i.e. Set 3), were seen on Day 1. On this test, only the reactivation + new learning group showed a memory impairment (i.e. lower recognition scores) compared to all other groups. Thus, Wichert et al. indeed showed that reactivation followed by interference affected consolidated memories.

Changing a memory in the lab via behavioural reconsolidation manipulations is a necessary first step in translating these findings to the psychological treatment of psychiatric disorders (Kindt & van Emmerik, 2016; Lane, Nadel, Greenberg, & Ryan, 2015). Theoretically, this could mean that in clinical practice patients first recall (i.e. reactivate) an emotionally distressing memory that is central to the psychiatric disorder. Subsequently they receive an appropriate behavioural intervention that modifies the memory. Afterwards, patients may suffer less from such an emotionally distressing memory (e.g. which may manifest itself as less intrusions in PTSD). Focusing on novel behavioural interventions is especially important, because recent studies show that frequently used pharmacological agents do not consistently affect reconsolidation in patients and healthy participants (Wood et al., 2015; for a meta-analysis see Lonergan, Olivera-Figueroa, Pitman, & Brunet, 2013). Given the potential of reconsolidation manipulations in psychological treatments, an important question is whether changing memories during reconsolidation by use of behavioural manipulations is a finding that can be replicated reliably.

Replication of reconsolidation findings is also crucial, because recently a substantial number of studies in psychological science failed to replicate the original results (e.g. Hagger et al., 2015; Maes et al., 2016; Maslany & Campbell, 2013; Matzke et al., 2015; Zwaan & Pecher, 2012; for a large-scale collaborative attempt to replicate 100 psychological experiments see Open Science Collaboration, 2015). This emphasizes the importance of independent replications; an appeal that has been made repeatedly in recent years to ensure the self-correcting nature of psychological science (e.g. Asendorpf et al., 2013; Koole & Lakens, 2012; Makel, Plucker, & Hegarty, 2012; Nosek, Spies, & Motyl, 2012; Pashler & Wagenmakers, 2012). In this spirit, we attempted to replicate Wichert et al.’s (experiment 1, 2013a) findings using similar procedures, manipulation, measures, and population.

We used Bayesian statistics to analyse the data, because these statistics allow for quantifying the amount of evidence in favour of the alternative hypothesis (H_1_), but also in favour of the null hypothesis (H_0_) (Dienes, 2016). Moreover, Bayesian statistics are increasingly used in the field of fear and trauma (e.g. Krypotos, Klugkist, & Engelhard, 2017; van de Schoot, Broere, Perryck, Zondervan-Zwijnenburg, & Van Loey, 2015). Contrary to Bayesian statistics, frequently used Null Hypothesis Significance Testing (NHST; e.g. Fisher, 1935) does not allow for a comparison of different hypotheses and only tests the evidence against the H_0_. As a consequence, *p*-values above .05 cannot be interpreted as evidence in favour of the H_0_. Yet, nine out of 10 replication studies are currently evaluated almost exclusively using NHST to test whether the effect is different from zero (i.e. testing evidence against, but never in favour of, H_0;_ Simonsohn, 2015).

Given that a number of researchers have expressed their concerns that a large number of published research findings may be false-positive findings (e.g. Ioannidis, 2005; Simmons, Nelson, & Simonsohn, 2011), testing whether the data are evidence either for H_1_ or H_0_ was our primary reason to use Bayesian hypothesis testing in this replication. We tested whether a memory impairment is indeed specific to the reactivation + new learning group compared to the three other groups (Reactivation, New Learning, and No Reactivation + No New Learning).

## 1. Method

### 1.1. Participants and design

Ninety-six students (48 males, 48 females; age: *M* = 21.2 years, *range* = 18–30) participated for course credit or a monetary compensation. Participants were excluded if they reported a current or chronic mental disorder, drug abuse or current treatment with medication, or if they were younger than 18 or older than 30 years. All participants gave written informed consent. The Ethical Committee of the Faculty of Social and Behavioral Sciences at Utrecht University (FETC15-001) approved this study.

**Figure 1.**
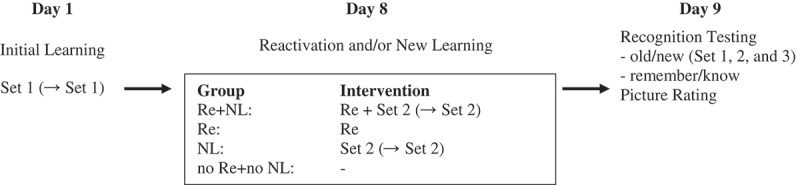
Experimental design. Day 1: initial learning of Set 1; Day 8: reactivation (Re) of initially learned pictures and/or new learning (NL) of Set 2 (depending on the condition). If participants did not reach the learning criterion on Day 1 or Day 8, the set was repeated once: (→ Set 1) or (→ Set 2). Set 3 was intermixed with Set 1 and Set 2 in the recognition test, and was a set of pictures that was never seen. Day 1 and Day 9 were identical for all participants.

Participants were randomly assigned to one of four groups with the restriction that men and women were equally assigned to each of the groups: (1) reactivation + new learning (Re+NL); (2) reactivation (Re); (3) new learning (NL); or (4) no reactivation + no new learning (no Re+no NL, see Figure 1).

### 1.2. Stimulus materials

Because the stimulus set from Wichert et al. (2013a) was relatively small (16 pictures), which makes ceiling effects in learning pictures likely, we used a larger stimulus set from Wichert et al. (2013b) (L. Schwabe, personal communication, 26 January 2015). We used three sets of 60 IAPS pictures (Lang, Bradley, & Cuthbert, 2005; 30 negative and 30 neutral) that were matched for valence and on arousal based on IAPS scores (see the Appendix for specific IAPS numbers). To validate these scores in our current sample, all participants rated one picture set at the end of the experiment. Valence and arousal were rated on a visual analogue scale ranging from 0 *neutral/not arousing* to 100 *negative/very arousing*. These ratings confirmed the original IAPS classification: negative pictures were rated as more negative (*M* = 66.01, *SD* = 14.09) than neutral pictures (*M* = 6.81, *SD* = 5.72), and they were rated as more arousing (*M* = 54.51, *SD* = 16.47) than neutral pictures (*M* = 9.57, *SD* = 7.11). Wichert et al. used neutral and negative pictures, but did not find any interactions effects. For replication purposes, we kept the materials’ valence matched with their study.

### 1.3. Procedure

In accordance with Wichert et al. (experiment 1, 2013a), testing was divided over three days: Day 1, initial learning; Day 8, reactivation and/or new learning; Day 9, recognition testing and picture rating (see Figure 1). A different set of pictures was used on each day; these sets were counterbalanced over the three days. We made small changes to the procedure of Wichert et al. (2013a) based on the study of Wichert et al. (2013b), which we detail below.

On Day 1, participants saw each picture from Set 1 presented individually on a computer screen. Picture presentation was the same as in Wichert et al. (2013b) who used a standardized viewing time of 2 s (1 s intertrial interval) per picture. After the picture presentation, participants verbally recalled as many pictures in as much detail as they could. There was no time limit for this free recall test. The experimenter scored the number of recalled pictures out of the participant’s sight and without giving any feedback. At least 20 out of 60 images needed to be correctly recalled in sufficient detail (e.g. ‘A man pointing a gun at a woman’ and not ‘A gun’) so that the picture could be uniquely identified. The number of pictures that needed to be correctly recalled was comparable with what participants recalled on average in Wichert et al. (2013b) on Day 1. If this criterion was not reached, presentation and recall was repeated once. Participants continued with the experiment regardless of the number of recalled pictures after the second recall. The learning session took approximately 25 minutes.

On Day 8, the experimental procedure was different for each group. Participants in the reactivation groups (Re+NL, Re) were brought back to the same spatial context as learning on Day 1 (see Hupbach, Hardt, Gomez, & Nadel, 2008). During reactivation, participants in these groups had two minutes to think back on the pictures that were presented on Day 1. Then, they verbally recalled the pictures they remembered. Directly after reactivation, participants in the Re+NL group were presented with new pictures from Set 2, following the same procedure and learning criterion as on Day 1. To control for specific effects of reactivation, the NL group learned and recalled Set 2 without reactivation of Set 1. This group recalled Set 2 in a spatial context that was different from Day 1. A final group (No Re+No NL) did not reactivate previously learned pictures or learned new pictures; they omitted a visit to the lab on Day 8.

On Day 9, all participants returned to the same lab as on Day 1 at approximately the same time as Day 8 (no more than 2 h before or after the time on Day 8). On average, the time between Day 8 and Day 9 was 24 h (*SD* = 1.04). On this day, participants completed a recognition test in which 180 pictures were randomly shown: 60 from Day 1, 60 from Day 8, and 60 never seen pictures (Set 3). In line with a two-step procedure (e.g. Eldridge, Sarfatti, & Knowlton, 2002), participants first indicated whether they had seen the picture on Day 1 by pressing a yes or no button. If participants pressed yes, they were required to judge whether they ‘remembered’ seeing the picture on Day 1 or whether they ‘knew’ so because of a feeling of familiarity (Yonelinas, 2002). We added the remember/know distinction to explore if the subjective feeling of remembering would be affected by a behavioural reconsolidation manipulation (see Schwabe, Nader, & Pruessner, 2013). After the recognition test, participants gave valence and arousal ratings for pictures from Set 1. Contrary to Wichert et al. (2013a), participants in our study rated valence and arousal at the end of the experiment to avoid confounds as a result of differences in encoding strength of the three sets (see also Wichert et al., 2013b). Finally, participants rated whether pictures from Day 1 were spontaneously or deliberately retrieved between Day 1 and Day 8 (i.e. strengthening of initial learning) and whether pictures from Day 1 were spontaneously or deliberately retrieved in the hours before the Day 8 appointment (i.e. reactivation opening the reconsolidation window). Ratings for all four questions were given on a 5-point Likert scale with labels: *never, rarely, sometimes, often*, and *very frequently*.

### 1.4. Data analysis

All data were analysed using the BayesFactor package (Morey & Rouder, 2015) in R (R Core Team, 2015). This package determines a Bayes Factor (BF) per requested test, which expresses the relative likelihood of the data under H_1_ and the H_0_. Data in favour of the H_1_ are presented as BF_10,_ which can be interpreted as the BF of H_1_ against H_0_. BF_01_ represents the reversed interpretation, where the evidence is in favour of the H_0_. These BF representations are used when Bayesian ANOVAs are performed. When the hypothesis is directional and one group is expected to perform better or worse than another group on a given variable, a Bayesian *t*-test is used. Here, the directional alternative hypothesis (e.g. A performs better than B) is compared to a complementary null hypothesis (e.g. A performs equal or worse than B). Because we hypothesized that memory change is specific to the Re+NL group, this group was always compared to the other three groups in follow-up analyses using Bayesian *t*-tests, whenever the Bayesian ANOVA indicated there was evidence in favour of the groups being different. As a prior we used a Cauchy distribution with scale *r* = 0.707, which is the standard (i.e. medium) prior in the BayesFactor package. Bayesian sensitivity analyses were performed with different priors to check the robustness of the results. Though the BF is a continuous scale, BFs can also qualified by categories of evidence to facilitate scientific communication (Jeffreys, 1961; Wetzels & Wagenmakers, 2012). BFs around 1 represent evidence neither in favour of H_1_ nor H_0_. BFs 1–3 represent anecdotal, 3–10 substantial, 10–30 strong, or 30–100 very strong evidence relative to the other hypothesis. A BF above 100 is interpreted as decisive evidence for a hypothesis relative to the other hypothesis. A BF_01_ of 2 therefore means that the data are twice as probable under H_0_ than under H_1_. Because a BF is relative, the BF for the other hypothesis is easily determined by dividing 1 by a given BF (e.g. if BF_01_ is 2, BF_10_ is 1 divided by 2, hence 0.5). Analyses for negative (BF_neg_), neutral (BF_neu_) or all pictures combined (BF_all_) are presented separately.

We first present the results on initial learning on Day 1, and memory reactivation and new learning on Day 8. However, the variable crucial to our research question is recognition accuracy on Day 9, and the further break-down of that variable in false alarms and hits (for recalled and non-recalled pictures).

## 2. Results

### 2.1. Initial learning on Day 1

Overall groups performed similarly and recalled a comparable total number of pictures (*M *= 25.34, *SD* = 5.34) during initial learning (BF_01 all_ = 11.13). Participants recalled more negative (*M *= 15.04, *SD* = 3.53) than neutral pictures (*M *= 10.3, *SD* = 3.07). Groups did not differ in their recall of negative pictures (BF_01 neg_ = 12.49); the evidence was indecisive for neutral pictures (BF_01 neu_ = 1). Participants required on average 1.74 trials to reach the learning criterion of 20 out of 60 images (see Table 1 for overall averages).

**Table 1.** Total Performance on Day 1 after initial learning, on Day 8 after reactivation and/or new learning, and on Day 9 for recognition testing. Means and standard deviations are presented.

Group	Day 1	Day 8		Day 9			
All	Pictures recalled after initial learning	Pictures recalled after Reactivation	Pictures recalled after New Learning	Recognition Accuracy (%)	Hits (%)	False Alarms from Day 8 (%)	Remember (%)
Reactivation + New Learning	25.96 (5.50)	20.29 (4.39)	24.63 (7.00)	63.96 (12.2)	71.87 (11.2)	11.74 (5.64)	50.39 (21.63)
Reactivation	24.83 (3.27)	17.92 (4.53)	-	73.19 (13.74)	79.51 (11.34)	6.25 (5.86)	47.4 (17.62)
New Learning	25.96 (6.33)	-	26.92 (5.12)	65.97 (16.62)	74.24 (14.3)	13.06 (13.02)	53.16 (17.42)
No Reactivation+ No New Learning	24.63 (5.92)	-	-	76.39 (10.98)	82.01 (11.14)	5.14 (6.43)	55.72 (19.3)

### 2.2. Memory reactivation and new learning on Day 8

The two groups that reactivated pictures from Day 1 recalled, on average, 19.1 pictures (*SD* = 4.57). Again, more negative pictures (*M *= 11.79, *SD* = 2.71) were recalled, than neutral pictures (*M *= 7.31, *SD* = 2.98). The analyses that compared the two reactivation groups show that the evidence does not unambiguously favour the null or the alternative hypothesis, regardless of stimulus valence (BF_01 all_ = 0.89, BF_01 neg_ = 1.20, BF_01 neu_ = 1.72). The two groups that viewed and recalled new pictures on Day 8 seemed to score similarly on the number of recalled pictures during new learning, regardless of stimulus valence (BF_01 all_ = 1.76, BF_01 neg_ = 3.00, BF_01 neu_ = 1.24), but the evidence in favour of the null hypothesis remains anecdotal. More negative pictures (*M *= 15.29, *SD* = 4.29) were learned than neutral pictures (*M *= 10.48, *SD* = 3.4). On average, these groups recalled 25.77 pictures (*SD* = 6.18).

### 2.3. Memory performance on Day 9

Our primary interest is recognition accuracy. This is the percentage of correctly recognized pictures from Set 1 (hits) minus the percentage of pictures from Set 2 or Set 3 that were incorrectly identified as being from Set 1 (false alarms), and the further break-down of that variable in hits and false alarms from Set 2. With this break-down, we are able to investigate whether general memory change in the different groups is the result of participants incorporating new information (i.e. false alarms of pictures from Set 2) into the original memory (Hupbach et al., 2007, 2008) or whether new information only impairs the memory, but is not incorporated into the original memory (i.e. a lower percentage of hits).^1^ See Table 1 for overall averages and Table 2 and 3 for averages separate for negative and neutral materials.

**Table 2.** Performance for Negative Pictures on Day 1 after initial learning, on Day 8 after reactivation and/or new learning, and on Day 9 for recognition testing. Means and standard deviations are presented.

Group	Day 1	Day 8		Day 9			
Negative	Pictures recalled after initial learning	Pictures recalled after Reactivation	Pictures recalled after New Learning	Recognition Accuracy (%)	Hits (%)	False Alarms from Day 8 (%)	Remember (%)
Reactivation + New Learning	15.17 (4.26)	12.42 (2.89)	15.37 (4.12)	67.50 (13.25)	76.94 (10.49)	12.92 (8.06)	63.40 (17.39)
Reactivation	14.88 (1.70)	11.17 (2.43)	-	78.89 (11.12)	86.11 (7.78)	7.08 (7.57)	63.18 (13.46)
New Learning	14.58 (3.48)	-	15.67 (3.36)	67.99 (16.18)	76.53 (13.42)	12.08 (12.50)	63.91 (13.59)
No Reactivation + No New Learning	15.54 (4.23)	-	-	80.69 (10.15)	86.39 (8.78)	5.00 (5.73)	69.95 (15.42)

**Table 3.** Performance for neutral pictures on Day 1 after initial learning, on Day 8 after reactivation and/or new learning, and on Day 9 for recognition testing. Means and standard deviations are presented.

Group	Day 1	Day 8		Day 9			
Neutral	Pictures recalled after initial learning	Pictures recalled after Reactivation	Pictures recalled after New Learning	Recognition Accuracy (%)	Hits (%)	False Alarms from Day 8 (%)	Remember (%)
Reactivation + New Learning	10.79 (2.83)	7.88 (2.77)	10.17 (2.73)	60.42 (16.76)	66.81 (15.62)	10.56 (6.50)	50.39 (21.63)
Reactivation	9.96 (2.88)	6.75 (3.14)	-	67.50 (19.18)	72.92 (17.29)	5.42 (6.20)	47.40 (17.62)
New Learning	11.68 (3.29)	-	11.25 (3.26)	63.96 (19.52)	71.94 (17.80)	14.03 (14.48)	53.16 (17.43)
No Reactivation + No New Learning	9.08 (2.93)	-	-	70.08 (13.80)	77.64 (15.15)	5.28 (9.68)	55.72 (19.30)

The analysis on recognition accuracy showed that the four groups differed (BF_10 all_ = 7.48). Interestingly, this effect was present in the negative pictures (BF_10 neg_ = 112.27), but not in the neutral pictures (BF_01 neu_ = 2.12). Follow-up analyses revealed that the evidence is prominently in favour of the hypothesis that the Re+NL group shows lower accuracy compared to the No Re+No NL group (BFs_10 all, neg, neu_ > 1398) and Re group (BFs_10 all, neg, neu_ > 7). There was only anecdotal evidence that the Re+NL group differs from the NL group (BF_10 all_ = 1.99, BF_10neg_ = 1.17, BF_10neu_ = 2.66).

A Bayesian ANOVA for the percentage of false alarms from Set 2 showed that the groups differed (BFs_10 all, neg, neu_ > 7.5). Follow-up tests revealed that – in accordance with our hypotheses – the Re+NL group had a higher percentage of false alarms than the No Re+No NL group (BFs_10 all, neg, neu_ > 38.9) and Re group (BFs_10 all, neg, neu_ > 85). However, the percentage of false alarms does not seem to differ between the Re+NL group and NL group, with the strongest evidence for neutral pictures (BF_01 all_ = 1.93, BF_10 neg_ = 1.49, BF_01 neu_ = 4.91). This suggests that the extent of memory updating is similar for the Re+NL and NL groups.

For the percentage of hits analyses show that groups differ. The effect seems to be specific for negative materials learned on Day 1 (BF_10 all_ = 2.75, BF_10 neg_ = 81.9, BF_01 neu_ = 2.67). Follow-up analyses show that hits are lower in the Re+NL group compared to the No Re+No NL group (BFs_10 all, neg, neu_ > 62) and Re group (BFs_10 all, neg, neu_ > 6.9). The analysis comparing the NL group and the Re+NL group revealed that the Re+NL group showed a reduced number of hits, but only for neutral pictures (BF_10 all_ = 2.52, BF_10 neg_ = 0.84, BF_10 neu_ = 4.84). However, this final result has to be interpreted with caution since the Bayesian ANOVA did not provide evidence for an overall group difference.

Because, according to reconsolidation, memories can only change when they are reactivated, we also performed analyses on hits for recalled pictures only. Re+NL and Re were directly compared, and indeed showed that Re+NL showed a reduced number of hits (BFs_10 all, neg_ > 3.28), yet not for neutral pictures (BF_01 neu_ = 5.54). Though, this comparison is interesting, a more crucial comparison would be between NL and Re+NL. Unfortunately, it is impossible to make this comparison with the current data. Alternatively, Wichert et al. (2013a) performed an analysis on hits for non-recalled pictures only in the Re+NL and Re groups. Because non-reactivated memories do not become labile, no change is expected. Hence, these two groups should perform similar. A direct comparison, however, revealed that Re+NL scores lower than Re (BF_10 all_ = 9, BF_10 neg_ = 96.39, BF_10 neu_ = 1.48).

Finally, we tested whether participants in the Re+NL group would display a reduced feeling of subjectively ‘remembering’ pictures from Day 1 compared to the other groups. Overall, analyses showed that there is substantial evidence that groups do not differ (BF_01 all_ = 4.6, BF_01 neg_ = 5.27, BF_01 neu_ = 7.03), which shows that the behavioural reconsolidation manipulation did not influence the self-reported source of memorizing: remembering vs. knowing.

### 2.4. Self-reported spontaneous and deliberate memory retrieval

At the end of the experiment, participants indicated retrospectively to what extent the pictures came or were brought to mind between Day 1 and Day 8. Analyses show substantial evidence in favour of the null hypothesis for either spontaneous (BF_01_ = 6.85) or deliberate strengthening (BF_01_ = 8.68) of initial learning. This suggests that, overall, memories of pictures were *rarely* strengthened spontaneously (*M *= 2.48, *SD* = 0.92), or deliberately (*M *= 2.02, *SD* = 0.95). Analyses for picture reactivation show similar results: substantial evidence for the null hypothesis either for spontaneous (BF_01_ = 8.05) or deliberate reactivation (BF_01_ = 8.41). Again, in general, memories were *rarely* recalled spontaneously (*M *= 2.51, *SD* = 1.17) or deliberately (*M *= 2.06, *SD* = 1.1).

## 3. Discussion

During reconsolidation, consolidated memories are temporarily sensitive to interventions that modify or update the original memory (e.g. Ågren, 2014). Memory modifications in the lab as a result of behavioural interventions are an important first step before translating these findings to clinical practice. Research into behavioural interventions during reconsolidation is especially important, because current psychological treatments are grounded in cognitive and behavioural interventions (e.g. cognitive-behavioural therapy; Rothbaum, Meadows, Resick, & Foy, 2000). Therefore, in the present study, we attempted to replicate one of the first studies using pictorial stimuli showing evidence for memory modification in episodic memory as a result of novel learning during reconsolidation, which controlled for reactivation and new learning (experiment 1, Wichert et al., 2013a). We were able to replicate some of the findings of the original study: relative to the groups Re and no Re+no NL, the group Re+NL, showed memory impairment and memory updating. However, we did not find that the crucial expected difference between Re+NL and NL alone was *substantially* supported by the data, for any of our dependent measures. Taken together with the analyses on hits for recalled and non-recalled pictures, the findings pose a challenge to predictions derived from reconsolidation theory.

The findings in our study can be explained without reconsolidation theory. The similarities between Re+NL and NL alone in our study may be the result of competition between items from the original memory and items from the new learning memory; an explanation that is in line with interference theory. The other groups, however, did not experience interference, because they did not learn something new on Day 8. Consequently, performing a recognition test for these groups should have been fairly easy. Moreover, the Wichert et al. (2013b) picture sets may have made performing the recognition test even more difficult for the new learning groups, because these picture sets had been matched not only on valence and arousal, but also on type of pictures in each set (e.g. each set contained a picture of a gun related event, a modern building, etc.), reducing the relative distinctiveness between sets. Furthermore, it is not surprising that interference was especially pronounced in neutral items (e.g. high false alarms), as non-emotional information is usually not as well remembered as emotional information is. Subsequently, this gives more opportunity for difficulties to arise in old/new recognition for neutral items (e.g. Dolan, 2002; Johnson, Hashtroudi, & Lindsay, 1993).

Our results can be explained by other theoretical accounts, such as interference theory, but it remains unclear why we did not observe effects in line with reconsolidation. Perhaps our failure to replicate the original results is related to the minor changes we made to the original design. To avoid ceiling effects we used three pictures sets of 60 pictures instead of 16 pictures, and we had participants rate one of these sets on valence and arousal at the end of the experiment instead of on Day 1. We also standardized picture-viewing time to 2 s on all days, while in the original the viewing time on Day 1 was determined by how long it took to perform picture ratings. Theoretically, any of these changes might be the sole or joint cause for the absence of effects in the current study. However, this seems rather unlikely, because all these changes were derived from another study by Wichert et al. (2013b) which also tested reconsolidation and showed that new learning after reactivation changed the original memory, even with all those modifications in the method. This suggests that the changes we made probably did not influence our results.

If small deviations from the original design cannot account for why our effects deviate from the original effects, then perhaps the presence of a moderating variable can. One straightforward explanation for lack of a clear difference between the Re+NL and NL groups is unintended reactivation in the NL group. Reactivation in the NL group would also have made the consolidated memory in this group sensitive to modification. Although, this is a possibility, we made particular efforts to avoid spontaneous reactivation in the NL group on Day 8. To achieve this, participants were tested by another experimenter and in a spatial context that was different from Day 1. This context switch was necessary, because memories are directly reactivated when participants return to the original learning context (Hupbach et al., 2008). Moreover, self-reports suggest that participants in all groups rarely reactivated pictures they originally learned before they came back to the lab, which renders it unlikely that unintended reactivation can account for the Re+NL group and NL group performing similarly.

It is unclear whether our replication is a false-negative finding or the finding from Wichert et al. (2013a) was a false-positive one. There is, of course, always a possibility of a non-replication, because a given number of replication attempts are inevitably doomed to be unsuccessful due to chance. However, our study joins a growing number of other studies showing failures to replicate the reconsolidation effect using behavioural manipulations (e.g. Golkar, Bellander, Olsson, & Öhman, 2012; Hardwicke, Taqi, & Shanks, 2016; Kindt & Soeter, 2013; Soeter & Kindt, 2011; Wichert et al., 2011) or pharmacological manipulations (e.g. Bos, Beckers, & Kindt, 2014; Wood et al., 2015). The study of Hardwicke et al. (2016) makes a particularly strong case against reconsolidation effects in humans, because it attempted to directly and conceptually replicate the results of a study that has been frequently referred to as a convincing demonstration of human reconsolidation in procedural memory (Walker, Brakefield, Hobson, & Stickgold, 2003), but was unsuccessful in seven experiments. Hardwicke et al. did not find any evidence for the impairment effects predicted by reconsolidation theory.

Still, lack of substantial support in our study clearly cannot nullify effects found in the study of Wichert et al. (2013a). Moreover, it is difficult to disregard memory reconsolidation as a whole based on a few studies that did not find reconsolidation effects; also given the considerable animal and human literature on this topic (e.g. Ågren, 2014; Schiller & Phelps, 2011). However, these studies can cast doubt on the reliability and effect sizes of previous results and subsequently on the theory itself, or at least on the boundary conditions and working mechanisms that are implied by the theory. By now, it is clear that reconsolidation is an intricate process, not merely dependent on reactivation followed by an intervention. It is a process that is suggested to be conditional on a number of boundary conditions, such as the context in which reactivation takes place (Hupbach et al., 2008), the original memory’s age and strength (Wichert et al., 2011), or whether something new is learned after reactivation (i.e. prediction error; Sevenster et al., 2012, 2013). The increasing number of boundary conditions raises the question whether other yet to be empirically uncovered boundary conditions may have dampened finding indisputable reconsolidation effects in this study.

Taken together, the current failed replication study highlights a number of critical points. First, the reconsolidation process may not be as reliable or as robust as previous reconsolidation studies have suggested. To increase our confidence in which findings are trustworthy, more direct or conceptual replications will be necessary (e.g. Nosek et al., 2012; Pashler & Wagenmakers, 2012). These replications will ultimately be essential in determining whether our and previous findings on reconsolidation are reliable. They will also be crucial for uncovering genuine boundary conditions of the reconsolidation process. The fact that we were unable to induce reliable change in relatively simple memories in well-controlled environments using behavioural reconsolidation manipulations, relates to a fundamentally important question for trauma-focused therapies like cognitive-behavioural therapy, EMDR, and imagery rescripting; how can complex and strong memories related to psychiatric disorders in real-life situations be changed reliably and safely? This remains an especially pertinent question since the boundary conditions that have been found, for instance the strength of the memory, differentiates memories created in the lab from memories related to psychiatric disorders, such as PTSD. Given that changing troubling real-life memories is one of the goals in clinical practice, it is crucial that future research on reconsolidation further advances our understanding, so that we will eventually be able to bridge the gap from lab research to clinical practice.

## Note

1. To foreshadow some of our findings: Bayesian sensitivity analyses were performed for all dependent variables of primary interest, including recognition accuracy, false alarms, hits (recalled and non-recalled), and remember percentage. Different priors (i.e. medium, wide, or ultra-wide) did not influence these results and therefore confirm that the reported results are robust.

## Highlights

- Consolidated memories can enter an unstable phase after reactivation before being restabilized (called ‘reconsolidation’).
- Wichert et al. (2013a) found that only reactivated episodic memories can be altered through learning new information.
- Replication is necessary before translation to potential clinical application.
- This study could not fully replicate Wichert et al. (2013a): memory change occurred after novel learning, regardless of prior reactivation of the original memory.
- The findings do not support that reconsolidation is the underlying mechanism.

## Appendix

IAPS numbers of pictures presented on Day 1, Day 8, and/or Day 9.

**Table:**

Set 1		Set 2		Set 3	
Neutral	Negative	Neutral	Negative	Neutral	Negative
7004	1200	2890	1274	9070	1201
7056	2981	5410	6570	2850	3064
7060	6313	2272	9265	5740	3051
7546	3100	7043	9042	7000	9592
7140	3130	7036	3071	7009	9220
7207	9423	7175	3225	7351	6312
2191	2703	7006	2205	7700	3530
2840	2710	7491	3500	9360	9330
2102	8485	7038	9435	7234	9910
7503	9911	5390	5973	7100	9421
7031	1270	2210	1220	2190	1275
7055	9561	7506	2811	1450	9420
7041	6315	2880	6940	5520	9405
7640	3068	7059	3110	7034	3230
7037	3140	7595	3080	7057	9415
2037	9425	7190	9410	7211	6530
7285	9040	7035	2717	7490	6250
2214	3180	5731	9428	7224	9611
8010	9120	9700	9520	7170	9921
7095	9830	5535	9320	7900	6821
7090	1301	2221	1525	2397	9560
7002	9800	8311	6210	7160	9433
7186	6560	1670	9600	7039	3400
7493	3550	7052	3150	7080	3160
7590	3170	7130	3016	7233	3300
2620	2683	7710	9570	7150	9041
5250	9253	7235	3350	7185	6212
2487	9432	5900	9810	7510	9301
2396	9926	7620	9000	7504	9181
7217	9001	7205	9290	7950	2751
